# High iNOS and IL-1β immunoreactivity are features of colitis-associated colorectal cancer tumors, but fail to predict 5-year survival

**DOI:** 10.48101/ujms.v128.10241

**Published:** 2024-01-02

**Authors:** Kajsa Björner, Wei-Na Chen, Venkata Ram Gannavarapu, Fredrik Axling, Miklos Gulyas, Mohammad Abdul Halim, Dominic-Luc Webb, Per M. Hellström

**Affiliations:** a Department of Medical Sciences, Gastroenterology and Hepatology Section, Uppsala University, Uppsala, Sweden; b Department of Surgical Sciences, Uppsala University, Uppsala, Sweden; c Department of Immunology, Genetics and Pathology, Uppsala University, Uppsala, Sweden

**Keywords:** Colorectal cancer, colitis-associated colorectal cancer, Interleukin-1β, inducible nitric oxide synthase, inflammatory bowel disease, tumor biology

## Abstract

**Background:**

Inflammatory bowel disease (IBD; mainly ulcerative colitis and Crohn’s disease) is associated with the development of colorectal cancer (CRC) referred to as colitis-associated colorectal cancer (CAC). In inflammatory flares of IBD, the production of luminal nitric oxide (NO) increases due to the increased inducible nitric oxide synthase (iNOS) activity in inflamed tissue. It is believed that iNOS parallels pro-inflammatory interleukin-1β (IL-1β). How these biomarkers relate to CAC pathogenesis or survival is unknown.

**Aim:**

The primary aim of this study was to investigate iNOS and IL-1β immunoreactivity in CAC tumors in comparison with CRC and normal colonic mucosa, and the secondary aim was to determine if immunoreactivity correlates with 5-year survival of CAC.

**Methods:**

Immunohistochemistry was performed on tissue sections as follows: CAC (*n* = 59); sporadic CRC (sCRC) (*n* = 12); colonic mucosa >2 cm outside sCRC margin (normal mucosa) (*n =* 22); paracancerous IBD (pIBD) (*n =* 12). The expression of iNOS and IL-1β was quantified separately for epithelium and stroma. Data were evaluated using the Mann-Whitney U-test and the log-rank test for 5-year Kaplan-Meier survival curves. Results were compared with online mRNA databases.

**Results:**

Immunoreactivity occurred predominantly in epithelial cells and to lesser extent in stroma. Compared with normal mucosa, immunoreactivity for iNOS (*P* < 0.01) and IL-1β (*P* < 0.005) was higher in CAC epithelium. In CAC stroma, iNOS immunoreactivity was lower than normal mucosa (*P* < 0.001), whereas IL-1β was higher (*P* < 0.05). Immunoreactivity differences of iNOS or IL-1β among CAC patients failed to correlate with 5-year survival. These findings were supported by online mRNA databases.

**Conclusion:**

Consistent with high NO production in IBD, there is more iNOS in CAC epithelium, albeit not in stroma. This immunoreactivity difference exists for IL-1β in both epithelium and stroma. The intervention of arginine or iNOS activity for CAC chemotherapy is not straightforward.

## Introduction

Inflammatory bowel disease (IBD) has increased in Sweden over the past century. IBD is subdivided into ulcerative colitis (UC), Crohn’s disease (CD), and unclassified (IBD-U). Data published a decade ago for the Uppsala region reported that yearly incidence of new cases of UC was 20/100 000, and that of CD was 9.9/100 000 (1, 2). Since 1970, the total population here has steadily risen by 5–10% every 5 years and is currently ~400 000, amounting to roughly 100 new IBD cases annually, some of whom went on to develop colorectal cancer (CRC). IBD is associated with increased risk of CRC (3–6), justifying surveillance for cancer. Higher risk has been associated with long duration of IBD, extensive disease, active inflammation, and primary sclerosing cholangitis (PSC) (7–10). For research purposes, IBD patients who develop CRC are subclassified as ‘colitis-associated colorectal cancer’ (CAC). Findings from epidemiological studies exploring parameters such as standardized mortality rate (SMR) in IBD vary but generally show higher mortality rates than for the background population (11). An important contributor to elevated SMR is CAC (11, 12). This implies that chronic inflammation in IBD is mitogenic and drives tumorigenesis.

Elevated rectal nitric oxide (NO) can be found in active colitis (13). Free diffusion across membranes and the unpaired electron of this gaseous-free radical account for DNA double strand breaks thought to drive tumorigenesis. An important biochemical pathway for this *in vivo* is the reaction of NO with superoxide, forming highly reactive peroxynitrite, which oxidizes purine moieties within nucleosides and DNA (14–16). In addition to cells of the innate immune system, intestinal epithelial cells also appear to be able to generate NO. In support of this, human intestinal epithelial cells are responsive to IL-22 (17), a cytokine shown to drive inducible NO synthase (iNOS) expression, increasing nitrotyrosine and DNA breakage within colonic mucosal epithelial cells (18). Methotrexate induces reversible senescence in human sCRC cells with cell cycle-dependent changes in iNOS expression and the inhibition of NO production (19). Reversibility of methotrexate-induced senescence is thought to underlie its ineffectiveness in treating CRC. Similarly, iNOS-depleted mice develop more polyps and dysplasia than those with functional iNOS (20). NO production in normal physiology is tightly regulated. Any deviation in NO production (higher or lower) is potentially tumorigenic. Thus, enforcing normal NO production early in IBD may prevent CAC. These studies further suggest that once tumors form, interventions targeting reduction in NO will not be tumoricidal. Although it is tempting to hypothesize that severity of inflammation in IBD correlates with CAC survival, it is unclear whether iNOS immunoreactivity, normally regarded as a pro-inflammatory biomarker, would correlate with cancer survival.

CRC prognosis is based on features of carcinoma cells and tumor stroma. Tumor stroma, consisting of fibroblasts, inflammatory cells, and endothelial cells, is important for tumor progression (21, 22). Crosstalk between carcinoma and stroma cells can enhance tumor formation and survival (23). Both iNOS and IL-1β have been implicated in this signaling (24, 25).

IL-1β is a highly pleiotropic cytokine with several downstream effects, for example, in acute and chronic inflammation and in recruitment and activation of lymphocytes and NK-cells. IL-1β is not intracellularly active and needs to be secreted to become active (26). Elevated mucosal IL-1β mRNA and protein expression have been reported in IBD (27, 28). Small amounts of IL-1β induce a specific immune response with limited inflammation, but the overexpression of IL-1β gives an extensive inflammation and tissue damage and enables tumor invasiveness (29, 30). The Cancer Genome Atlas (TCGA) database identified high IL-1β expression to be correlated with longer survival (31). Like iNOS, IL-1β seems capable of exerting a dual role in tumorigenesis and tumor defense. Both inflammatory proteins are biochemically connected insomuch as IL-1β activation of NF-ĸB leads to iNOS expression. To our knowledge, concomitant immunoreactivity has not been investigated in CAC tumors.

The primary aim of this study was to investigate iNOS and IL-1β immunoreactivity in CAC tumors compared with CRC tumors and non-cancerous colon mucosa. As a secondary aim, the role of these two biomarkers in 5-year survival of CAC was investigated. The test hypothesis was that iNOS and IL-1β are higher in CAC, and immunoreactivity predicts survival.

## Methods

### Study subjects and samples

Patients with IBD and CRC (i.e. CAC) registered between 1970 and 2020 were identified from inpatient and outpatient registries. Inclusion criteria for CAC patients were as follows: a confirmed diagnosis of IBD; the diagnosis based on established clinical, endoscopic, and histological criteria (32); and concomitant colorectal adenocarcinoma with available blocks of cancer tissue. Exclusion criteria were of registry diagnosis of IBD or CRC that could not be confirmed in medical records. Sections from formalin-fixed paraffin-embedded blocks of cancerous tissue from 59 identified CAC subjects were used for histopathological examination and iNOS and IL-1β immunohistochemistry (IHC). This was compared to several different tissues stained according to the same protocol: 1. Normal colonic mucosa >2 cm outside CRC boundary (normal mucosa) (*n =* 22); 2. Paracancerous IBD mucosa <2 cm outside the CAC boundary (pIBD) (*n =* 12); 3. Cancer tissue from sCRC (*n =* 12). Exclusion criteria for CRC tissue were radiation therapy prior to surgery. Findings from normal mucosa were further verified in biopsies with no morphological evidence of inflammation from age-matched patients with Parkinson’s disease (*n =* 10). The study protocol was approved by the Central Ethics Review Board (Dnr Ö 21-2012) and Stockholm and Regional Ethics Review Board (2017/219), Uppsala. All living subjects gave their written consent.

### Immunohistochemistry

IHC for the quantification of iNOS (rabbit polyclonal, 1:200 (final 20 µg/ml) ab3523, Abcam, Cambridge, UK) and IL-1β (rabbit polyclonal, 1:300 (1.7 µg/ml), ab9722, Abcam, Cambridge, UK) was performed on separate slides. Sections of 4 µm thickness were cut from paraffin-embedded blocks, deparaffinized, rehydrated, and microwaved for antigen retrieval. Endogenous peroxidases were blocked with H_2_O_2_ and sodium azide. Slides were incubated with primary antibodies overnight at 4°C. Antigen-antibody complex was visualized with 3,3´-diaminobenzedine (DAB) using a commercial horseradish peroxidase kit (Detection-Envision FLEX system; DAKO #K800021-2, Agilent, Santa Clara, CA, USA), followed by counter-staining in Mayer’s hematoxylin. This was followed by dehydration and organic mounting (Pertex®, Histolab Products AB, Gothenburg, Sweden).

### Scoring

For each subject, a representative part of each tissue section was selected for immunoreactivity scoring. Quantification of immunoreactivity was calculated separately for iNOS and IL-1β by multiplying percentage of positive cells, 0, <5%; 1, 5–25%; 2, 26–50%; 3, 51–75%; 4, >75%, and by staining intensity, 0, no staining; 1, weak; 2, moderate; 3, strong, creating a value of 0–12. Epithelium and stroma were scored separately. All sections were examined by two independent investigators (KB and MG). When observations resulted in disparate scores, joint examinations were carried out to reach consensus.

### Statistics

Non-parametric statistics were used for all calculations. Statistical differences between two groups were determined with the Mann-Whitney U-test. Differences between several groups were first analyzed with the Kruskal-Wallis test, followed by the Mann-Whitney U-test. To compare survival rates against immunoreactivity, subjects were stratified for iNOS or IL-1β score. ‘High’ immunoreactivity was defined as a score above or equal to median scoring value. ‘Low’ immunoreactivity was defined as a score below median. Kaplan-Meier curves were plotted for the separate high and low immunoreactivity groupings of iNOS and IL-1β, followed by statistical evaluation with the log-rank test. Correlations were calculated using the Spearman’s rank test. Statistical operations were done using Statistica software, version 13 (TIBCO Software Inc., Palo Alto, CA, USA).

## Results

### Patient characteristics

A total of 59 CAC patients were identified by cross-analysis of electronic medical records from the Uppsala University Hospital. Patient characteristics are presented in Table 1. IBD subtype distribution was UC (*n =* 39), CD (*n =* 18), and IBD-U (*n =* 2). Of these, 17 had pediatric onset of IBD. Extensive disease was the most common phenotype, occurring in 12 of 18 (67%) CD patients, 26 of 39 (67%) UC patients, and both IBD-U cases. Among all 59 patients, seven (18%) had PSC, five with UC, one with CD, and one with IBD-U.

**Table 1:** Clinical characteristics of CAC cohort.

	All	CD	UC	IBD-U
*n* (%)	59 (100)	18 (31)	39 (66)	2 (3)
**Sex**				
Female	17 (29)	5 (28)	11 (28)	1 (50)
Male	42 (71)	13 (72)	28 (72)	1 (50)
**Age at IBD diagnosis**				
Mean (years)	28	29	30	24
Median (years)	27	31	27	24
<18	17 (29)	6 (33)	11 (28)	
18 to <40	28 (47)	7 (39)	19 (49)	2 (100)
40 to <60	11 (19)	4 (22)	7 (18)	
≥60	3 (5)	1 (6)	2 (5)	
**Age at cancer diagnosis**				
Mean (years)	52	54	51	52
Median (years)	51	56	50	52
<18	1 (2)		1 (3)	
18 to <40	14 (24)	4 (22)	10 (26)	
40 to <60	25 (42)	8 (44)	16 (41)	1 (50)
≥60	19 (32)	6 (33)	12 (31)	1 (50)
**Stage at cancer diagnosis**				
Stage 1	12 (20)	2 (11)	9 (23)	1 (2)
Stage 2	15 (25)	4 (22)	10 (26)	1 (2)
Stage 3	19 (32)	7 (39)	12 (31)	
Stage 4	11 (19)	4 (22)	7 (18)	
Unknown	2 (3)	1 (6)	1 (3)	
**Extension of IBD**				
Proctitis	3 (5)		3 (8)	
Leftsided	8 (14)		8 (21)	
Extensive	40 (68)	12 (67)	26 (67)	2 (100)
Ileocecal	3 (5)	3 (17)		
Unknown extent	5 (8)	3 (17)	2 (5)	
**Localization of cancer**				
Rectum	15 (25)	6 (33)	9 (23)	
Left colon	11 (19)	2 (11)	8 (21)	1 (50)
Transverse colon	4 (7)	1 (6)	3 (8)	
Right colon	22 (37)	5 (28)	16 (41)	1 (50)
Multiple locations	4 (7)	2 (11)	2 (5)	
Anastomosis	2 (3)	2 (11)		
Unknown location	1 (2)		1 (3)	
**Concomitant cancer location**				
Yes	46 (78)	14 (78)	30 (77)	2 (100)
No	8 (14)	1 (6)	7 (18)	
Unknown	5 (8)	3 (17)	2 (5)	

CD: Crohn’s disease; UC: ulcerative colitis; IBD: inflammatory bowel disease; IBD-U: unclassified IBD.

### iNOS and IL-1β immunoreactivities are higher in CAC

**Figure 1:**
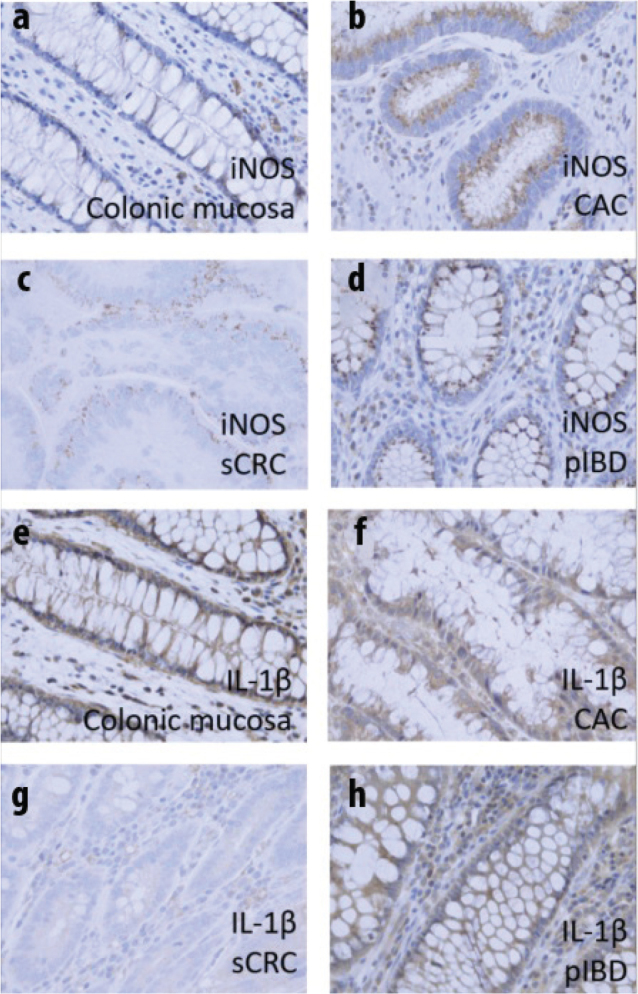
Immunoreactivity of iNOS in normal mucosa (a), CAC (b), sCRC (c), and pIBD (d). Immunoreactivity of IL-1β in colonic mucosa (e), CAC (f), sCRC (g), and pIBD (H). IL-1β has a strong diffuse staining in epithelium as well as stroma.

Figure 1 shows representative images from the various tissues. Negative control experiments revealed that the presence of primary antibody was required to obtain DAB staining. Figure 1a–d exemplifies iNOS immunoreactivity in normal mucosa (a), CAC (b), sCRC (c), and pIBD (d). In iNOS panels, perinuclear localization in epithelial cells is evident. Figure 1e–h exemplifies diffuse immunoreactivity of IL-1β in normal mucosa (e), CAC (f), sCRC (g), and pIBD (h).

Immunoreactivity of iNOS was found in epithelial cells of all 59 tissue sections from the 59 CAC patients studied, with immunoreactivity scores ranging from 1 to 12. There was polarized and apparently perinuclear localization of iNOS in most cells in the epithelium in highly differentiated cancer and non-cancerous tissue. When iNOS immunoreactivity was found in cancerous tissue of low differentiation, the epithelial architecture was disrupted, and the location of iNOS was less distinct.

Immunoreactivity scores for iNOS in normal mucosa, CAC, pIBD, and sCRC are presented in Table 2. Comparing all four groups, a significant difference was found for iNOS in the epithelium (*P* < 0.05) and stroma (*P* < 0.0001). Immunoreactivity of iNOS was found in all sections of normal mucosa, CAC, pIBD, and sCRC. Immunoreactivity of iNOS in the epithelium was significantly higher in CAC compared to normal mucosa. There were no differences between immunoreactivity scores of iNOS in the epithelium of normal mucosa, pIBD, and sCRC.

**Table 2:** Differences in iNOS and IL-1β immunoreactivity in normal mucosa, CAC, sCRC, and pIBD. Data are immunoreactivity scores, median (IQR).

	Epithelium	*P* (vs. NM)	Stroma	*P* (vs. NM)
**iNOS**				
Normal mucosa	4 (4–8)	N.A.	3.5 (2–4)	N.A.
CAC	8 (6–9)	***P* < 0.01**	2 (1–3)	***P* < 0.001**
sCRC	4.5 (2–9)	*P* = 0.52	1 (0.5–2)	***P* < 0.0001**
pIBD	6 (5–8)	*P* = 0.41	2.5 (1–4)	*P* = 0.26
**IL-1β**				
Normal mucosa	5 (4–8)	N.A.	3.5 (2–4)	N.A.
CAC	8 (6–9)	***P* < 0.05**	4 (3–6)	***P* < 0.05**
sCRC	4 (4–4)	*P* = 0.12	3 (2–4)	*P* = 0.64
pIBD	8 (8–8)	*P* = 0.09	6 (4–7)	***P* < 0.005**

iNOS: inducible nitric oxide synthase; NM: normal mucosa; CAC: colitis-associated cancer; sCRC; sporadic colorectal cancer; pIBD: paracancerous IBD mucosa.

Bold values indicate statistical significance.

In stroma, iNOS immunoreactivity was found in 52 of the 59 CAC patients with scores ranging from 1 to 4. In normal mucosa, iNOS immunoreactivity was found in stroma of all 22 patients. pIBD was found in 11 out of 12 patients, and sCRC was found in 9 of 12 patients. Normal mucosa had significantly higher iNOS immunoreactivity in stroma than in CAC (*P* < 0.001) or sCRC (*P* < 0.0001) (Table 2).

IL-1β immunoreactivity was found in epithelium of all tissue sections from CAC (ranging 3–12), pIBD, and sCRC patients. In normal mucosa, IL-1β immunoreactivity was found in 21 of 22 patients, located throughout the cytosol of nearly all epithelial cells. Immunoreactivity of IL-1β in epithelium was higher in CAC than in normal mucosa (*P* < 0.05). There were no differences of IL-1β in normal mucosa compared with pIBD or sCRC (Table 2).

In stroma, IL-1β immunoreactivity was found in all sections from patients with CAC (ranging 1–9), and in normal mucosa, it was found in pIBD and sCRC patients. Compared with normal mucosa, IL-1β immunoreactivity in stroma was higher in CAC (*P* < 0.05) and pIBD (*P* < 0.005) patients. There was no difference of IL-1β in control tissues compared to sCRC (Table 2).

**Figure 2:**
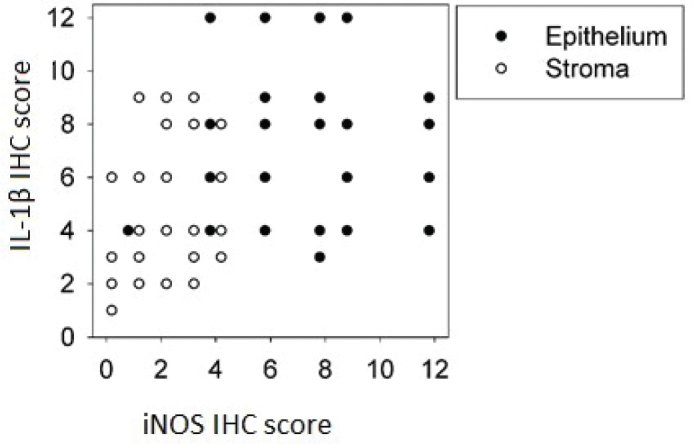
Correlations of iNOS and IL-1β IHC scores in epithelium and stroma in CAC. *n =* 59 (one slide per patient). Epithelium *R* = 0.12, *P* = 0.345 (NS); stroma *R* = 0.36, *P* < 0.01.

The quantified immunoreactivity in CAC showed no correlation between iNOS and IL-1β in epithelium (*r* = 0.12, *P* = 0.35), whereas in stroma, a weak correlation between iNOS and IL-1β was found (*r* = 0.36, *P* < 0.05) (Figure 2). In normal mucosa, immunoreactivity scores of iNOS and IL-1β did not correlate in epithelium (*r* = 0.01, *P* = 0.97) or stroma (*r* = −0.14, *P* = 0.53).

Biopsies from colon mucosa of subjects with Parkinson’s disease were immunoreactive for iNOS as well as IL-1β in both epithelium and stroma.

### iNOS and IL-1β immunoreactivities fail to predict outcome

**Figure 3:**
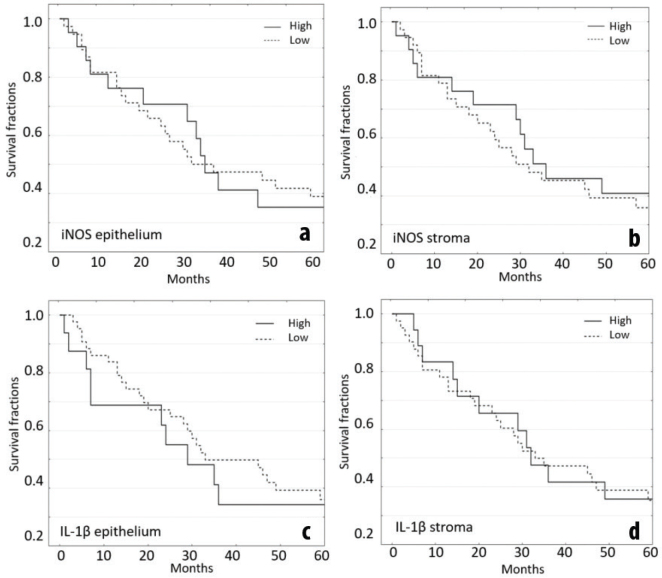
Kaplan-Meier survival plots for CAC stratified by iNOS or IL-1β in epithelium or stroma. (a) Epithelium score for iNOS, high (*n =* 38) and low (*n =* 11). (b) Stroma score for iNOS, high (*n =* 38) and low (*n =* 21). (c) Epithelium score for IL-1β, high (*n =* 43) and low (*n =* 16). (d) Stroma score for IL-1β, high (*n =* 41) and low (*n =* 18). ‘High’ was defined as score above or same as median; ‘low’ was defined as score below median.

CAC survival rates did not differ between patients with high versus low iNOS immunoreactivity scores in epithelium (Figure 3a, *P* = 0.94) or high versus low iNOS scores in stroma (Figure 3b, *P* = 0.68). Similarly, CAC survival curves of high versus low IL-1β scores did not differ for epithelium (Figure 3c, *P* = 0.63) or stroma (Figure 3d, *P* = 0.93). CAC survival time did not correlate with iNOS immunoreactivity scores in epithelium (*r* = 0.15) or stroma (*r* = −0.04) or with IL-1β immunoreactivity in epithelium (*r* = 0.02) or stroma (*r* = 0.13). Combining immunoreactivity scores of iNOS and IL-1b in epithelium as well as stroma also did not result in any correlation. Hazard ratios (HR) for all survival comparisons would, therefore, not be statistically different from 1.

### IHC findings are supported by online mRNA data

Online databases were searched for mRNA data to explore consensus with the present IHC results. Dataset GSE4183 (33) was used for establishing baseline mRNA expression and statistical comparisons between *NOS2A* and *IL1B* expression in normal colon, CRC, and IBD biopsies. Survival differences for low versus high *NOS2A* and *IL1B* mRNA expression were explored in public mRNA datasets for CAC- and CRC-derived samples. Dataset GSE3629 (34) was identified as having comparison mRNA data for CAC versus CRC, but there was no survival data. TCGA Pan-Cancer Analysis Project (35, 36) has survival data for a large number of samples defined as colon adenocarcinoma (COAD) and rectum adenocarcinoma (READ), but IBD cases within were not documented. Therefore, a CAC gene profile was generated. A set of 40 genes that discern CAC from UC without cancer (34) were used to screen another mRNA dataset for overlapping significant expression differences between IBD and CRC (33). This resulted in a profile of seven genes: *LRP6*, *BTN3A1*, *GBP4*, *GBP1*, *CYSLTR1*, *GBP5*, and *SLA*. These were used to isolate likely CAC samples in the TCGA pan-cancer cohort. Out of 588 adenocarcinoma samples, 22 matches for CAC were identified. These were used for comparison of *NOS2A* and *IL1B* mRNA expression in CAC versus CRC (*n =* 566) and survival analysis. Details from these searches appear in Supplement 1. Findings are summarized in Table 3. *NOS2A* and *IL1B* genes were expressed in normal colon. Median expression of both genes was higher in IBD. Both medians were higher in CAC than CRC, with *NOS2A* reaching significance. Survival in CAC for lower versus higher expression did not reach significance for either gene. Data in the online databases were generally in line with the IHC findings.

**Table 3:** Expression and survival data from online mRNA databases.

Median mRNA expression	Normal	IBD	CRC^a^	CAC^b^	*P*		
					IBD vs.	CRC vs.	CRC^a^ vs.
					Normal	Normal	CAC^b^
*N*			566	22			
***NOS2A*** (counts)	-	-	506.0	1415.7			0.0141
***IL1B*** (counts)	-	-	252.7	405.4			0.1507
*N*	8	14	15				
***NOS2A*** (RE)	251.9	2102.9	340.6	-	0.0009	0.1464	
***IL1B*** (RE)	311.6	2242.7	2131.0	-	0.0003	0.0009	
							
**Survival based on mRNA**							
	CRC^a^		CAC^b^				
*N* (low|high)	283	283	11	11			
***NOS2A***							
HR (low/high)		0.651		0.770			
Logrank P		0.027		0.730			
***IL1B***							
HR (low/high)		0.775		1.042			
Logrank P		0.180		0.960			

HR: hazard ratio; counts (normalized counts); RE: relative expression; CRC: colorectal cancer; COAD: colon adenocarcinoma; READ: rectum adenocarcinoma; CAC: colitis-associated colorectal cancer; Normal: normal colon; IBD: inflammatory bowel disease; P-value, Mann-Whitney U-test.

a CRC counts (normalized counts) = combined COAD and READ datasets from TCGA Pan-Cancer Atlas.

b Proposed CAC cases from COAD and READ datasets. See also Supplement 1.

## Discussion

IBD patients suffer from higher risk of CRC than the background population. This is especially true for younger patients. In Sweden, the median age for the diagnosis of colon cancer was 74 years between 2007 and 2022 (37), and 71 years for rectal cancer between 1995 and 2022 (38). The median age of diagnosis in our study is markedly lower (51 years). Survival of CAC patients remains poor despite lower stage at diagnosis achieved by surveillance. This implies that earlier age of CAC diagnosis relative to CRC is not an artefact of earlier detection due to surveillance of IBD patients. As NO is regarded as a biomarker of inflammatory activity in colonic disease, immunoreactivity of iNOS and IL-1β (e.g., as by promoting iNOS expression) was hypothesized to predict CAC prognosis. Immunoreactivities of iNOS and IL-1β were, indeed, higher in CAC than the other groups. However, differences in immunoreactivity did not correlate with 5-year CAC survival. Apparently, higher iNOS and IL-1β in CAC tumors represent features typical of the preexisting background IBD but are not variables in CAC aggressiveness.

This study covered patients diagnosed with CAC over a 50 year period. The findings are consistent with elevated iNOS and IL-1β in tumor growth. Nath et al. (39) reviewed current understanding that NO produced by iNOS in cancer cells promotes tumor growth, whereas NO produced by iNOS in TAMs is tumoricidal. These effects might change over time in response to different stimuli. Expression and degradation rates of iNOS as well as NO production and downstream signaling could all depend on timepoint in tumor progress. The spread in iNOS immunoreactivity scores allows for this to be possible. Differences in immunoreactivity cannot discern changes in expression from changes in degradation and may not be proportional to enzyme activity or products (e.g., NO vs. direct S-nitrosation) that depend on other chemical variables and binding with other proteins. The lack of significant correlation of iNOS or IL-1β immunoreactivity with survival could indicate that iNOS and IL-1β were sufficiently elevated in most CAC patients such as to have saturated any effects on survival.

Tumor-associated macrophages (TAMs) are important for immunological tumor defense. Local cytokines stimulate differentiation to type 1 (M1) or type 2 (M2) TAMs (40). M1 is antitumoral, whereas M2 has pro-tumorigenic properties. The M1 strongly expresses iNOS, which generates high NO concentrations with deleterious effects on tumor cells. There were insufficient numbers of macrophages in the present sections to quantify M1 versus M2 TAMs, so this was not pursued. However, this study found that normal colonic mucosa also expresses iNOS and hence must be a substantial source of NO. Immunoreactivity of iNOS and IL-1β correlated in stroma but not epithelium. This could be attributed to interaction between iNOS and IL-1β in TAMs within the stroma as previously reported (39). IL-1β is part of the inflammatory response of M1 macrophages. Secreted form of IL-1β can diffuse into the tumor microenvironment and has been discussed to potentiate molecules from malignant stroma cells that promote invasiveness and metastasis (30).

Most biomarkers for the outcome of adenocarcinoma used in histology are related to the adenoma-carcinoma pathway and show mutations in oncogenes such as KRAS, NRAS, BRAF, APC, and DDF (41). Ki-67 is used as a cellular marker for proliferation (42). Cancer development in IBD differs from the main population. The inflammation-dysplasia-carcinoma pathway APC dysregulation and KRAS mutations are less frequent than in CRC (43). NOD-like receptor pyrin domain-3 (NLRP3) inflammasome is upregulated in CRC. When NLRP3 was inhibited in a xenograft mouse model, IL-1β and IL-18 decreased (44). Inflammation is thought to be a driver for the development of CAC and many cancers in general. Because luminal NO is elevated in colitis, this study instead focused on tumor immunoreactivity of IL-1β (a known inducer of iNOS) and iNOS. Factors such as increased peroxynitrite production may well provoke tumorigenesis in IBD. Once this advances to CAC, other factors, such as mutations in oncogenes, are more predictive of survival.

In normal mucosa and highly differentiated tumors, iNOS immunoreactivity usually had a peri-nuclear localization, whereas in low-differentiated cancerous mucosa, iNOS immunoreactivity was usually distributed diffusely throughout the cytosol. The present IHC scoring, which only quantified iNOS immunoreactivity, covered the entirety of the analyzed tumor tissues, so did not factor in subcellular localization. The fact that normal colonic mucosa also had iNOS immunoreactivity confirms findings by others (45–47) that described either local expression in apical cytoplasm facing the lumen or less distinctly in epithelium. Differences in localization could conceivably involve different iNOS binding proteins and targets for S-nitrosation and even different relative amounts of liberated NO versus direct S-nitrosation. Biochemical consequences in CAC are unclear to us.

Immune checkpoint inhibitors (ICIs) are a new, game changing treatment for many patients, but still some cancers resist even this treatment. It has been shown *in vitro* that an NO donor can help overcome immune resistance for ICI (48). More traditional cytostatic agents can have better effect when coupled with an NO donor (49). For the time being, limitations in applying NO or NO-modified drugs have insufficient specificity, despite systemic toxicity. There is no consensus on ICI treatment for CAC. Expert opinion is that it can be beneficial if the colitis is well treated (50).

This study demonstrated iNOS and IL-1β expression in cancerous as well as non-cancerous tissue and in inflamed tissue as well as non-inflammatory tissue. IL-1β expression was higher in CAC epithelium as well as stroma compared to normal mucosa. The iNOS immunoreactivity, on the other hand, was only more pronounced in CAC epithelium. However, survival curves were not separable by differences in iNOS or IL-1β immunoreactivity. The fact that so many cancer cells displayed immunoreactivity for both proteins implies that a positive feedback mechanism may exist in which IL-1β is secreted by the cancer cell and binds to the receptor on the same cell, activating NF-ĸB, which, in turn, activates iNOS expression (51). The survival findings prevent a simple understanding of what impact intervening in such a loop (e.g., arginine metabolism or NOS inhibition) would have for CAC patients.

## Strengths and limitations

IHC was carried out using established methods and clearly visualized the two biomarkers against reasonably low background. However, IHC represents momentary immunoreactivity without any means of pairwise comparisons in same individual over time. Because iNOS has different products and protein interactions, it is possible that survival is associated with some aspect of iNOS activity not revealed by immunoreactivity. Because IL-1β is a cytokine (i.e. secreted into circulation), immunoreactivity in tumors cannot unambiguously differentiate cells that express and secrete IL-1β from those that only have IL-1β receptor and internalize it.

The CAC sample size (*n =* 59) covers all identifiable cases in the past 50 years from the local population meeting inclusion/exclusion criteria. Extracted data therefore reflect actual fallout of clinical work in this select group of patients. Expanding the study by increasing the sample size would risk additional insecurities of appending non-local clinical data. If a much larger sampling is needed to reach significance, then an effect at the level of immunoreactivity would be small and inconsistent and, therefore, of poor predictive value for any individual CAC patient. Resources should focus on other variables.

## Conclusion

Immunoreactivities of iNOS and IL-1β are increased in CAC, which should primarily be considered features of preexisting background chronic inflammatory processes in IBD. Elevated luminal NO concentration reported in colitis likely derives from supraphysiological mucosal iNOS expression with greater enzyme activity, chronically increasing peroxynitrite, thus increasing cancer risk.

## Data Availability

Data that are used in the results and conclusions in this study can be provided upon reasonable request.
